# A Tool Set to Map Allosteric Networks through the NMR Chemical Shift Covariance Analysis

**DOI:** 10.1038/srep07306

**Published:** 2014-12-08

**Authors:** Stephen Boulton, Madoka Akimoto, Rajeevan Selvaratnam, Amir Bashiri, Giuseppe Melacini

**Affiliations:** 1 Departments of Biochemistry and Biomedical Sciences, McMaster University, 1280 Main Street West, Hamilton, Ontario, L8S 4M1, Canada; 2 Chemistry and Chemical Biology, McMaster University, 1280 Main Street West, Hamilton, Ontario, L8S 4M1, Canada

## Abstract

Allostery is an essential regulatory mechanism of biological function. Allosteric sites are also pharmacologically relevant as they are often targeted with higher selectivity than orthosteric sites. However, a comprehensive map of allosteric sites poses experimental challenges because allostery is driven not only by structural changes, but also by modulations in dynamics that typically remain elusive to classical structure determination methods. An avenue to overcome these challenges is provided by the NMR chemical shift covariance analysis (CHESCA), as chemical shifts are exquisitely sensitive to redistributions in dynamic conformational ensembles. Here, we propose a set of complementary CHESCA algorithms designed to reliably detect allosteric networks with minimal occurrences of false positives or negatives. The proposed CHESCA toolset was tested for two allosteric proteins (PKA and EPAC) and is expected to complement traditional comparative structural analyses in the comprehensive identification of functionally relevant allosteric sites, including those in otherwise elusive partially unstructured regions.

Physiological function and homeostasis are tightly regulated by allostery. Allosteric regulation is also often exploited pharmacologically for enhancing target selectivity^1^,^2^,^3^,^4^,^5^,^6^,^7^,^8^,^9^,^10^,^11^. One of the most common mechanisms of allosteric regulation relies on the coupling of binding and conformational equilibria, which is modeled by a four-state thermodynamic cycle (Figure 1A). For example, in one of the prototypical allosteric systems, *i.e.* the regulatory subunit of Protein Kinase A (PKA R), activation is controlled by a dynamic equilibrium between inactive and active conformations that differ not only at the binding site of the allosteric effector, *i.e.* cAMP, but also at remote loci essential for inhibition of the catalytic subunit (Figure 1A)^7^,^8^,^12^,^13^. The allosteric effector cAMP binds with higher affinity to the active *vs.* the inactive conformation of PKA R. Because of the active *vs.* inactive state selectivity of cAMP, binding of cAMP to the regulatory subunit shifts the conformational equilibrium towards the active state (Figure 1A), weakening the association of the regulatory and catalytic subunits and releasing kinase inhibition. Allosteric cycles (Figure 1A) provide therefore a simple but effective thermodynamic model of how ligands allosterically regulate remote inhibitory sites.

In order to manipulate the thermodynamics of allostery for therapeutic purposes, it is essential to fully map at atomic resolution the active *vs.* inactive state differences, which will be collectively referred to here as ‘allosteric networks'. When each discrete functional state (*e.g*. inactive and active) is structurally homogeneous, *i.e.* adopts a well-defined and distinct structure as determined through classical structure determination methods^14^,^15^,^16^, comparative structural analyses effectively map allosteric networks of interactions that link distal protein sites. For example, in the cAMP binding domain (CBD) of PKA R, which is composed of an α- and a β-subdomain, active *vs.* inactive conformational differences are observed primarily in the α-subdomain and in the cAMP binding motif known as the phosphate binding cassette (PBC) (Figure S1)^17^,^18^. Upon cAMP binding, the PBC and the C-terminal helices shift inwards towards the β-subdomain, while the N-terminal helical bundle (NTHB) moves away from the β-subdomain (Figure S1B). In this case, the comparative structural analysis of R bound to either the kinase subunit (C) or to cAMP is effective in revealing the cAMP-dependent allosteric networks within the globular CBDs of PKA.

While comparative structural analyses are an invaluable approach for the elucidation of allosteric networks, a growing body of evidence indicates that allostery relies not only on structural changes, but also on modulations of dynamics^19^,^20^,^21^,^22^,^23^,^24^. In addition, critical inhibitory sites under allosteric control are often found in partially unstructured regions, such as flexible linkers that remain elusive to classical structure determination methods^12^,^13^,^14^,^19^,^25^. Two examples that illustrate the importance of dynamics and flexible linkers in allostery are provided by the Exchange Protein directly activated by cAMP (EPAC) and PKA R. In EPAC1, a region known to mediate multiple inhibitory cAMP-dependent interactions with the catalytic domain was found to be subject to only minimal cAMP-dependent structural changes^24^,^26^,^27^,^28^. However, cAMP binding to EPAC1 causes an enhancement of dynamics in this region, weakening the underlying auto-inhibitory interactions by imposing an entropic penalty^24^,^26^. In PKA R, the comparative analysis of crystal structures could not reliably identify allosteric sites in a dynamic linker critical for kinase inhibition, because electron density was either missing or affected by crystal packing^12^,^17^,^18^. However, the linker was later found to elicit state selective interactions that allosterically couple it to cAMP^12^. For both EPAC and PKA R, these otherwise elusive dynamic allosteric sites were detected using an alternative approach known as the CHEmical Shift Covariance Analysis (CHESCA)^12^,^25^. In addition, CHESCA has been applied to other systems, revealing amino acid networks underlying enzyme catalysis and inhibition, which have been confirmed by independent mutational analyses, and shows promise for *in vivo* applications^12^,^25^,^29^,^30^,^31^,^32^,^33^,^34^,^35^.

The CHESCA method is particularly effective in mapping functionally relevant allosteric sites within dynamic and partially unstructured regions, which are common in signalling systems but often escape detection through classical structure determination methods^3^,^9^,^12^,^23^,^25^,^29^,^30^,^31^,^32^. CHESCA relies on the covariance analysis of NMR chemical shifts to identify and functionally categorize allosteric networks of residues eliciting concerted responses to a small library of analogs of the allosteric effector ligand. The analogs feature covalent modifications that perturb the non-covalent interactions anchoring the endogenous allosteric effector (*i.e.* cAMP) to its receptor (*i.e.* PKA RIα) and typically include reverse-agonists (*i.e.* Rp-cAMPS, Figure 1B), antagonists, partial (*i.e.* 2′-OMe-cAMP, Figure 1B) and full agonists (*i.e.* Sp-cAMPS, Figure 1B). These functionally diverse ligands are utilized under fully saturating conditions to effectively lock the inactive *vs.* active equilibrium at different degrees of activation. {^15^N, ^1^H}-HSQC spectra are then acquired for the allosteric protein under investigation either in the *apo* form or saturated by each selected ligand (Figure 1C). To reduce the dimensionality of the HSQC chemical shifts, the nitrogen and proton chemical shifts of each residue (δ_N_ and δ_H_, respectively) are linearly combined as: 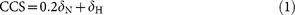
where CCS is the compounded chemical shift. When the inactive *vs.* active exchange is fast in the NMR chemical shift time scale, as often the case^12^,^25^, the chemical shifts observed for residues sensing exclusively the allosteric conformational equilibrium are linear weighed averages between those of the pure active and inactive states. Under these conditions, the modulation of the inactive *vs.* active equilibrium by the ligands in the CHESCA library results in residue-specific CCS changes that are linearly correlated (Figure 1D)^18^,^25^ Hence, linear inter-residue pairwise CCS correlations (IPCs, Figure 1D) serve as effective signatures for residue pairs exhibiting a concerted response to the perturbations implemented by the CHESCA library. In this respect, IPCs provide the foundation for the systematic elucidation of allosteric networks^25^.

Another critical feature of the CHESCA method is that the point-distribution in the IPCs provides a means to assign a function to the residue networks identified through CHESCA. For instance, networks defined by IPCs similar to the one shown in Figure 1D, in which inhibited forms (*i.e.* *apo* or bound to reverse-agonists and antagonists) are segregated from the active forms (*i.e.* bound to agonists), are assigned an allosteric function. Whereas networks featuring IPCs in which the separation is between *apo* and bound forms, irrespective of the degree of activation, are assigned a primarily binding function^12^,^25^. An exhaustive map of allosteric and binding networks requires therefore the identification of all possible IPCs defined by a given library of perturbations (*i.e.* ligand analogs and/or mutations).

In order to systematically identify all possible residue pairs involved in linear IPCs, the correlation matrix (**R**) is computed. **R** is the correlation matrix of **M** transpose, where **M** is a matrix obtained by compiling the observed CCS values, in which rows correspond to residues and columns to ligands in the CHESCA library. In the original implementation of CHESCA, denoted here as CHESCA-SL, the **R** matrix was utilized to identify residue clusters based on single linkage hierarchical clustering^12^,^25^,^31^, an agglomerative algorithm in which a single linear IPC is sufficient for assigning a residue to a cluster, *i.e.* cluster growth relies on local criteria^12^,^25^,^31^. Single linkage hierarchical clustering is effective in exhaustively reconstructing allosteric networks, minimizing false negatives even when all possible linear IPCs are not experimentally observed. However, single linkage methods are also known to be biased by chaining effects that lead to false positives^36^,^37^,^38^,^39^.

Here, we show how false positives arising from the use of single linkage clustering in CHESCA-SL are identified and minimized. The identification of false positives relies on cross- checking the CHESCA results through an independent method to analyse chemical shifts, *i.e.* the chemical shift projection analysis (CHESPA)^40^. The CHESPA is a simple vector analysis of the (^1^H, ^15^N) chemical shifts for three states, typically the *apo*, the allosteric effector-bound and an analog-bound form (Figure S2). Hence, CHESPA, unlike CHESCA, alone is not suitable to identify allosteric networks, but it is useful to monitor at residue-resolution the response to selected ligands in the CHESCA library and cross-check the CHESCA-derived clusters for false positives, *i.e.* residues with analog-responses differing from the majority of the remaining residues in the same cluster. We also show that once false positives are identified through CHESPA, they can be minimized through the use of a clustering algorithm complementary to single linkage, *i.e.* complete linkage agglomerative clustering (CHESCA-CL).

Our results indicate that CHESCA-CL significantly reduces the number of false positives, but does not completely eliminate them. We show that the residual false-positives arise from the degeneracy intrinsic to the combination of (^1^H, ^15^N) chemical shifts into a single CCS, as defined in equation (1). The residual false-positives are eliminated by a modified CHESCA scheme based on the covariance analysis of separate ^1^H and ^15^N chemical shifts. The combination of complete-linkage and separate ^1^H and ^15^N chemical shifts results in a robust algorithm (CHESCA-I) for the reliable identification of allosteric networks. The robustness of CHESCA-I was confirmed by the overall agreement with the allosteric networks independently mapped through the inactive *vs.* active comparative structural analysis of well-folded globular domains. Furthermore, for less structured and dynamic regions, CHESCA-I preserves the ability to identify otherwise elusive functional allosteric sites. Overall, the proposed algorithms (CHESCA -SL, -CL and -I) define a CHESCA tool set for reliably mapping allosteric networks and we provide a ‘user guide' flow chart for the effective implementation of this CHESCA tool set, through which potential false positives and false negatives are detected and minimized.

## Results and Discussion

### The Chemical Shift Projection Analysis (CHESPA) Reveals False Positives in CHESCA-SL

**Figure 2:**
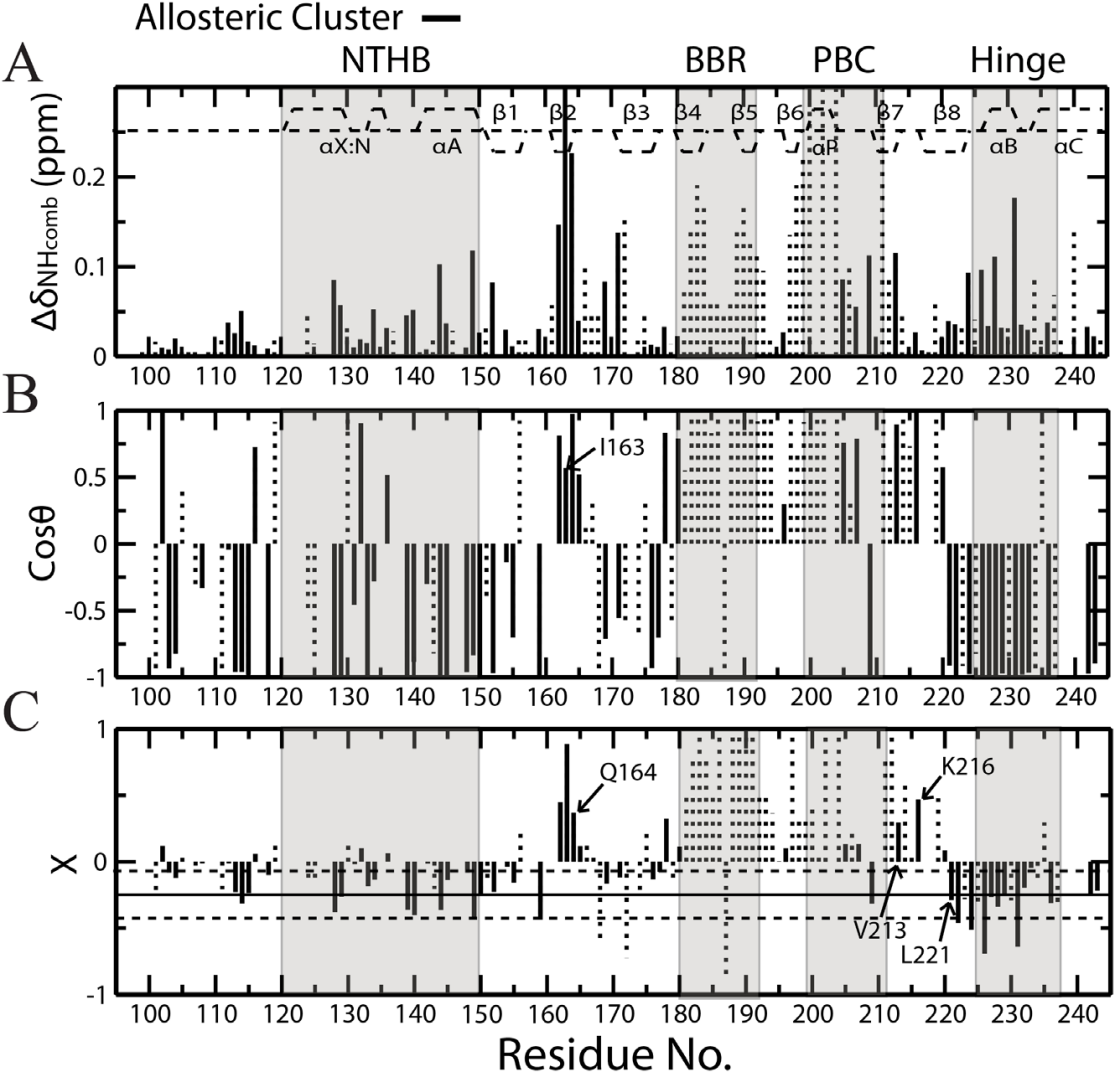
The chemical shift projection analysis (CHESPA) of R_p_-cAMPS (Rp) bound PKA RIα CBD-A. (A) The Δδ_NHcomb_ *vs.* residue plot for R_p_-cAMPS bound *vs.* *apo* PKA RIα (91-244). Solid black lines represent residues belonging to the allosteric cluster identified from the CHESCA-SL analysis, while the Δδ_NHcomb_ values of other residues are reported as dotted vertical bars. The secondary structure of PKA is shown as dashed lines at the top of the plot. (B) The cosθ *vs.* residue plot. The θ angle is defined in Figure S2. (C) Plot of fractional activations (X) *vs.* residue. Fractional activations are defined in Figure S2. The solid horizontal line represents the average of all the negative X values with adjacent horizontal dashed lines corresponding to the average +/- one standard deviation. Selected residues are highlighted.

The CHESPA analysis of R_p_-cAMPS (or Rp in short) was used to identify false positives in the allosteric clusters defined through CHESCA-SL as applied to RIα 91-244, which spans the critical CBD of PKA. Rp was chosen over the other analogs in the CHESCA library since it is a reverse agonist^41^,^42^,^43^. Therefore, residues sampling primarily the allosteric inactive *vs.* active equilibrium are expected to exhibit ppm changes reflecting an opposite shift in the activation equilibrium relative to cAMP (fractional activation X < 0; Figure S2), whereas residues affected by Rp binding but not allosteric conformational changes would experience chemical shift changes similar to cAMP (X > 0) as well as unique ppm shifts influenced by the replacement of the equatorial phosphate oxygen with a bulkier sulphur atom in Rp (*i.e.* NNEs). The residue-specific X values observed for Rp in PKA RIα 91-244 are shown in Figure 2. As expected, the CHESPA analysis of Rp results in a splitting of chemical shift changes between those that shift in a direction similar to cAMP (X > 0) and those that shift in the opposite direction (X < 0; Figure 2). The negative X values are observed primarily for residues in the α-subdomain, which was previously predicted to play an integral role in the allosteric activation of PKA, while the positive X values are mainly localized in the β-subdomain, a region that contains two important cAMP binding elements, the base binding region (BBR) and the phosphate binding cassette (PBC) (Figure S1).

Residues from the CHESCA-SL allosteric network are highlighted in Figure 2 as solid vertical lines to cross-check whether the CHESCA-SL analysis could distinguish reliably between allosteric and binding elements. Allosteric residues are expected to sense the same conformational equilibrium and hence to share similar fractional activations (X). While this is proved true for the majority of the residues in the allosteric cluster identified through CHESCA-SL (Figure 2C), a subset of residues within the same cluster (*e.g.* β-core residues 162–165, 178, 213, 216) exhibit positive fractional activations (Figure 2C). Among these, some correspond to marginal X values close to zero, suggesting that they are within the noise of the CHESPA analysis, but other residues, such as I163, Q164 and K216, feature significant fractional activations (Figure 2C) and clearly capture false positives of the CHESCA-SL analysis, which is based on single linkage clustering, a method notorious for the presence of chaining effects.

### One Source of False Positives in CHESCA-SL is the Single-Linkage “Chaining” of Weakly Correlated Residues

**Figure 3:**
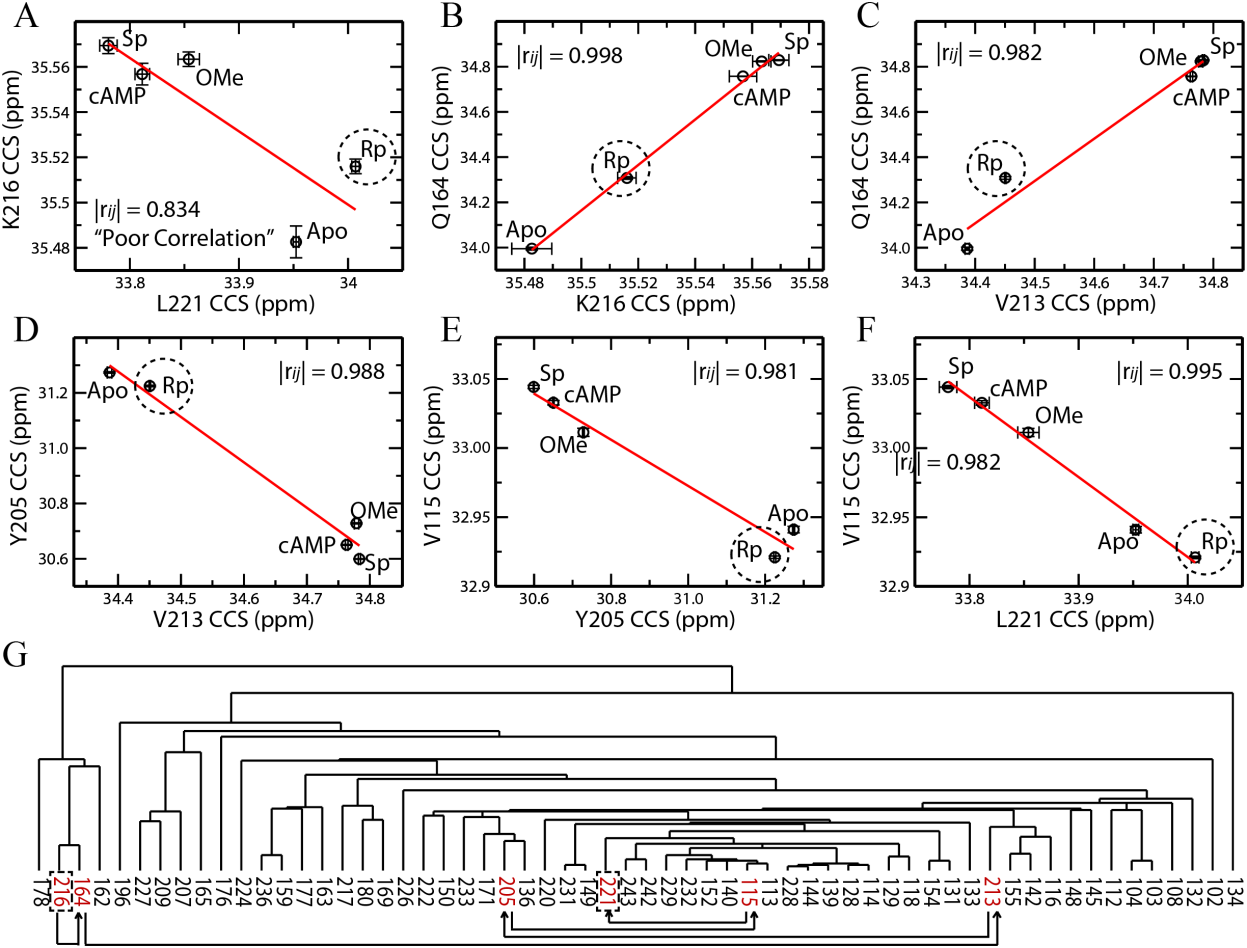
Analysis of inter-residue CCS pairwise correlations reveals the “chaining effect” of single-linkage agglomerative clustering. (A) Inter-residue pairwise correlation between L221 and K216. Despite the poor K216 *vs.* L221 correlation, single-linkage clustering assigns L221 and K216 to the same cluster because of a chain of strong correlations that links these two residues together, as shown in panels (B–F). (G) The single-linkage dendrogram for the allosteric residue cluster reveals the branch locations of residues in panels (A–F) (red residues). The black arrows outline the chain of correlations connecting K216 and L221 (dashed rectangles).

We hypothesized that residues with opposite fractional activations (X) are clustered together by CHESCA-SL because of an inherent chaining property of the single linkage hierarchical agglomerative clustering method utilized in CHESCA-SL. Single-linkage clustering links two clusters together if there is a high correlation between any of the residues within either of the clusters^36^,^37^. Even if the majority of correlations for residues between those two clusters are poor, a single high correlation will still cause the two clusters to be linked together. For example, residues K216 and L221 belong to the same allosteric cluster as defined by CHESCA-SL, but exhibit fractional activations with opposite signs (Figure 2C) and hence are very poorly correlated, as shown in the IPC of Figure 3A. The correlation coefficient between K216 and L221 is 0.83, which is significantly lower than the 0.98 cutoff typically used in CHESCA-SL^12^,^25^. Furthermore, Figure 3A shows that along the K216 axis the Rp state is positioned roughly midway between the *apo* state (inactive) and the three other *holo* states cAMP, Sp and OMe (active), whereas along the L221 axis it is the *apo* state that is found between the Rp and the three active states. Hence, the example illustrated in Figure 3A reveals that single-linkage may cluster together two residues, such as K216 and L221, with markedly different responses to the ligands in the CHESCA perturbation library.

We hypothesized that the partitioning of two functionally distinct residues, such as K216 and L221, to the same cluster arose from a chain of strong correlations that links together residues for which the direct correlation is very weak. We further expected to see in this chain of correlations very subtle, but consistently incremental shifts in the distributions of states that would explain the large net difference in distributions that are observed between K216 and L221. As expected, a chain of strong pairwise correlations linking the weakly correlated K216 and L221 residues was identified (Figure 3B–G). This chain begins with the K216-Q164 pair (Figure 3B), then continues with the Q164-V213, V213-Y205 and Y205-V115 sequential correlations (Figure 3C–E) and eventually ends with the V115-L221 IPC (Figure 3F,G). At each step in this chain, the Rp state is subject to a subtle progressive shift towards the *apo* state, until it eventually crosses it (Figure 3B–F). The distribution of states at the beginning of the chain, with Rp midway between the *apo* and *holo* states, is reminiscent of a correlation between residues affected by binding and nearest neighbour perturbations unique to the interactions with Rp. In contrast, the distribution of states at the end of the chain more closely reflects the expected positions for allosteric residues. It is notable that a similar shift is observed for the partial agonist 2′-OMe-cAMP (or OMe in short) bound state^41^,^44^,^45^. Near the beginning of the chain, its position is close to the other bound states, such as cAMP and the full agonist Sp-cAMPS (Sp), but as the chain progresses, it slowly shifts towards the *apo* state. This observation corroborates that residues at the beginning of the chain play a role in binding, while those near the end report primarily on the allosteric equilibrium. Overall, the example in Figure 3A illustrates the possibility that single linkage clustering chains together within a single cluster residues with divergent responses to the CHESCA library (*i.e.* chaining effect). Hence, the chaining effect results in false positives within the allosteric cluster identified by CHESCA-SL.

### Complete-Linkage Clustering Overcomes the Chaining Effect

**Figure 4:**
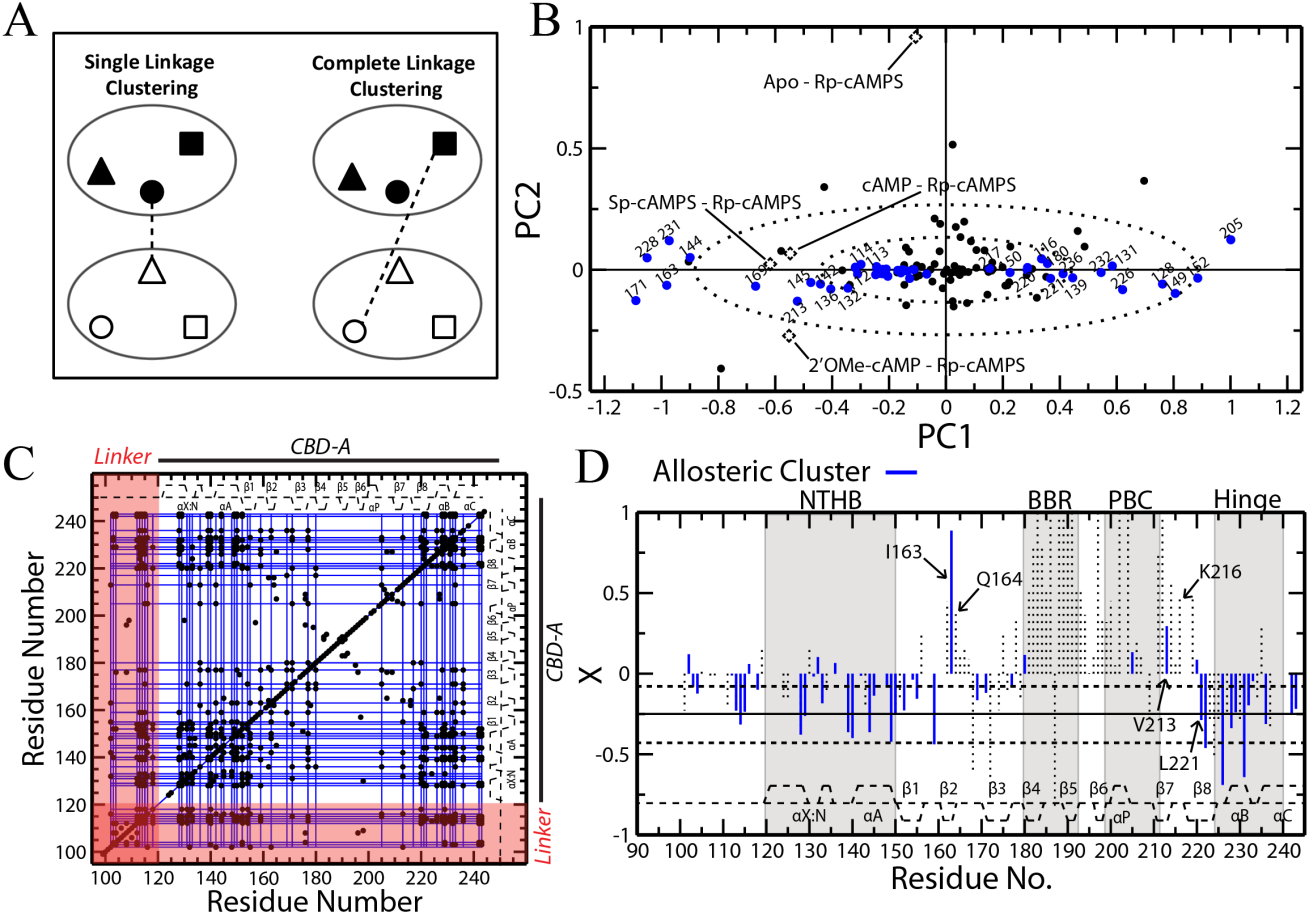
Complete-linkage agglomerative clustering maps allosteric networks without “chaining effects”. (A) Schematic comparison of single *vs*. complete linkage clustering. The shapes (triangle, circle and square) represent residues within existing clusters (large ovals) and the dashed line depicts the method by which the two clusters are linked. (B) Singular value decomposition (SVD) analysis of the combined chemical shifts of PKA RIα (91-244). Loadings are shown as black dashed diamonds and scores are shown as circles. Blue scores represent residues from the reconstructed allosteric cluster determined by complete-linkage clustering (Figures S3 and S4). The dashed ovals correspond to the standard deviations of PC1 and PC2. (C) The correlation matrix for PKA RIα (91-244) with complete-linkage clusters shown as blue lines. Only correlations with |r*_ij_*| ≥ 0.98 are shown (black dots). The secondary structure is displayed as dashed lines along the top and side of the plot. (D) A plot of Rp-bound fractional activations similar to Figure 2C, but with residues from the complete-linkage cluster shown as blue lines. Selected residues are labelled, including I164 and V213 that exhibit positive X values unlike the majority of the residues in the allosteric cluster identified through complete linkage.

To overcome the chaining effect caused by single-linkage clustering, we considered other types of clustering methods, such as complete linkage clustering. Complete linkage clustering examines the correlations of every pair of residues between groups and, unlike single linkage, will only link groups together if the lowest correlation coefficient among all pairs is above a designated cutoff^46^,^47^. This ensures that all residues within a given cluster are highly correlated with each other. Due to the high stringency of this method, the number of false positives in the correlations is expected to be considerably reduced relative to single linkage. Hence, we re-analyzed using complete linkage clustering the chemical shift data of PKA RIα previously utilized for CHESCA-SL^12^. Figure S3 shows the dendrogram representing the complete-linkage agglomerative clustering of PKA RIα (91-244). Figure S3 shows that the stringency of complete linkage fragments the single linkage clusters into sub-clusters with reduced size due to the sparse nature of the **R** matrix. For instance, using a correlation coefficient cutoff of 0.98, as for the single-linkage of CHESCA-SL^12^,^25^, the maximum cluster size is nine residues, almost one order of magnitude less than the maximum cluster size obtained through single-linkage (*i.e.* ~60 residues; Figure 3G)^12^. However, complete linkage generates multiple clusters with more than three residues (clusters I-VII, Figure S3 and S4A), which share similar distributions of their functional states (*i.e.* active *vs.* inactive), as proven by the corresponding state dendrograms (Figure S4B). Furthermore, all the residues included in the smallest dendrogram branch that spans clusters I-VII (blue box in Figure S3) exhibit singular value decomposition (SVD) scores aligned along the same principal component (PC), as shown in Figure 4B.

The SVD analysis offers an independent approach to separate residues that contribute to each of the major equilibria (*i.e.* binding and allosteric)^25^. The positions of the loadings in the PC plot reveal the functional role of residues spaced along a given axis. For example, in Figure 4B the loadings progress along PC1 from Sp, cAMP and 2′OMe to *apo* and Rp. This is the pattern expected for the allosteric equilibrium, thereby assigning to PC1 and to all residues with scores spaced along it a role in allosteric activation. In this respect, it is remarkable that all the amino acids within the selected branch of the complete-linkage dendrogram (blue box in Figure S3) correspond to scores that are aligned along PC1 with minimal variation along PC2 (Figure 4B), suggesting they are all associated to a similar allosteric function.

Based on the combined SVD (Figure 4B) and state dendrogram analyses (Figure S4B), the fragmented sub-clusters generated by complete-linkage were reassembled into a single allosteric cluster (Figure S3, blue box), which will be referred to here as the CHESCA-CL allosteric cluster. The protocol for reconstructing the CHESCA-CL allosteric cluster is summarized in Figure S5 and the corresponding allosteric network is depicted in Figure 4C as a grid of blue lines overlaid to the correlation matrix **R**. Figure 4C also illustrates that despite the added stringency of complete-linkage clustering, the previously identified critical allosteric sites in the flexible linker region spanning residues 100–120 are still captured. Finally, the allosteric residues from complete-linkage clustering were mapped on the fractional activation plot of Rp-bound PKA to verify that there is a significant reduction in the number of false positives (blue lines in Figure 4D).

**Figure 5:**
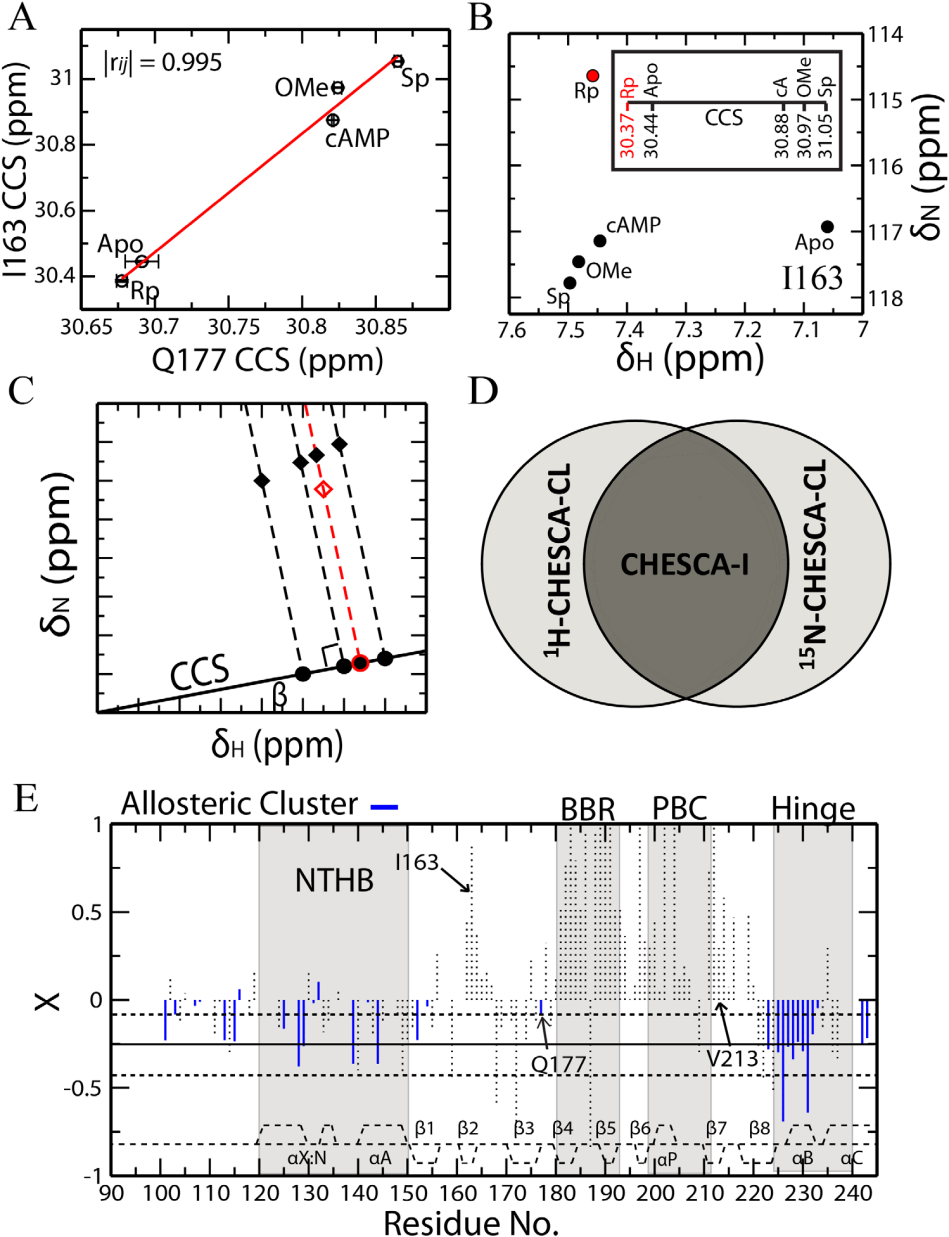
Potential artefacts arising from the use of the combined chemical shift (CCS) projection and an alternative CHESCA algorithm to account for them. (A) Selected inter-residue pairwise CCS correlation between two residues, I163 and Q177, with different fractional activations for the Rp-bound state. (B) HSQC cross-peak positions for residue I163. The CCS values of the five states are displayed in the insert. (C) Illustration of the similarity between the use of CCS and the projection of 2D (^1^H, ^15^N) cross-peaks into a single axis rotated by an angle β (equation 2). Different 2D (H,N) cross-peaks (black filled and red open diamonds) may result in similar CCS values (circles). (D) Scheme for a CHESCA approach designed to circumvent the projection compression effect (“CHESCA-I”). Two independent CHESCA-CL implementations are applied to the nitrogen and proton chemical shifts using a 0.95 |r*_ij_*| cutoff value (H- and N-CHESCAs) and only the residues that are conserved between the two complete-linkage clusters generated by the H- and N-CHESCAs are included in the functional network. (E) Rp-bound fractional activations for PKA RIα (91-244) with residues from the CHESCA-I method shown as blue lines. Solid and dashed horizontal lines are defined as in Figure 2C.

Figure 4D shows that, as expected, the number of residues with positive fractional activations was greatly reduced. For instance, Q164 and K216, which exhibit positive fractional activations and were classified as allosteric by single linkage clustering (Figure 2C), are now correctly excluded from the allosteric cluster defined through complete linkage (Figure 4D). However, there are still a few residues with positive fractional activations that are assigned to the allosteric cluster by CHESCA-CL (Figure 4D). The most significant of these is residue I163, with a positive fractional activation of ~0.9. To determine why this correlation remained, its IPCs were examined (Figure 5A), but surprisingly the IPCs for this residue matched the expected pattern that was observed for the majority of other complete-linkage allosteric residues, with both the inactive states (*i.e.* *apo* and Rp) well separated from the active ones (*i.e.* cAMP, 2′OMe, Sp) (Figure 5A). To understand this discrepancy between the CHESPA and CHESCA analyses, we turned to the other CHESPA parameter, the cosθ value (Figure 2B). Interestingly, for I163 the cosθ value is close to 0.5, revealing that the chemical shifts of these states were non-linear in the HSQC spectra (Figure 5B) and suggesting that the CHESPA *vs.* CHESCA discrepancy observed at the level of I163 arises from the use of the CCS (defined in equation 1) as opposed to the separate ^1^H and ^15^N ppm values in the covariance analysis.

### Another Source of False Positives Is the Combined Chemical Shift Projection Compression

Chemical shifts from different nuclei belonging to the same residue are often combined into a single linearly weighed average, in order to reduce the dimensionality of the chemical shift analysis and provide a single residue-specific descriptor utilized to map interactions at residue-resolution. Hence in our original CHESCA-SL implementation, we had calculated the combined ^1^H and ^15^N chemical shift using equation (1). In geometrical terms, the linear combination of equation (1) closely resembles a projection of the nitrogen and proton chemical shifts onto an axis rotated by an angle β relative to the original ^1^H axis of the ^1^H, ^15^N HSQC plane. This is appreciated by rewriting equation (1) as: 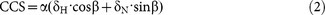
where α: 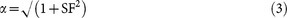
and: 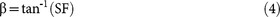
with SF being the scaling factor for the nitrogen chemical shifts, *i.e.* 0.2 in the case of equation (1), which results in β = 11.3° and α = 1.02. Interpreting the CCS equation (1) in terms of a projection onto a rotated axis visually illustrates how HSQC peaks that fall on the same projection line result in the same CCS (Figure 5C). In other words, it is possible that HSQC peaks with distinct chemical shifts lead to identical CCS values. We will refer to this type of CCS degeneracy as “projection compression”.

The CCS projection compression provides an explanation as to why I163, which exhibits positive fractional activations in the CHESPA analysis, was assigned by the CHESCA-SL and -CL schemes to the allosteric cluster populated primarily by residues with negative fractional activations (Figures 2C and 4D). The CHESPA relies on a vectorial analysis in a two-dimensional Cartesian coordinate system, thereby removing any effects from the CCS projection compression. On the contrary, the previous CHESCA implementations rely on a mono-dimensional CCS scale and thereby projection compression is a potential concern. For example, the HSQC peaks corresponding to the Rp and *apo* states of residue I163 are clearly well separated (Figure 5B), but they exhibit similar CCS values due to the projection compression (Figure 5B, insert). This results in a linear CCS distribution that fits the pattern expected for the allosteric cluster (Figure 5A).

To overcome the effect of the CCS projection compression, two independent CHESCA analyses were performed on separate ^15^N and ^1^H chemical shift matrices. Allosteric clusters were determined for each CHESCA analysis using complete-linkage similarly to the protocol discussed above and residues conserved in both the ^15^N and ^1^H–based allosteric clusters were selected (Figure 5D). The Rp fractional activations for these residues, defined as CHESCA-I allosteric ensemble, are displayed in Figure 5E as solid blue lines, showing that the residues with the largest positive fractional activations, such as I163 and V213, are now completely eliminated from the allosteric cluster (Figure 5E). However, there was also a significant reduction in the number of residues with negative fractional activations (Figure 5E) relative to the previous implementation of complete linkage using CCS (Figure 4D). One possible explanation is that inter-residue correlations were lost for those residues exhibiting linear HSQC variations largely along a single axis, either ^15^N or ^1^H, but not both. Thereby, these residues are detected by only the proton or nitrogen analysis and are consequently removed when collecting the residues conserved between the two analyses. It is therefore clear that, while the added value of this method is the stringent determination of pairwise correlations and the drastic reduction in the number of false positives, it also results in an increased number of false negatives and it should be used in conjunction with less stringent and complementary methods such as those relying on the combined chemical shifts (*i.e.* CHESCA-SL and -CL).

### Comparison of Structure-Based and CHESCA-Based Allosteric Networks within Folded Domains

**Figure 6:**
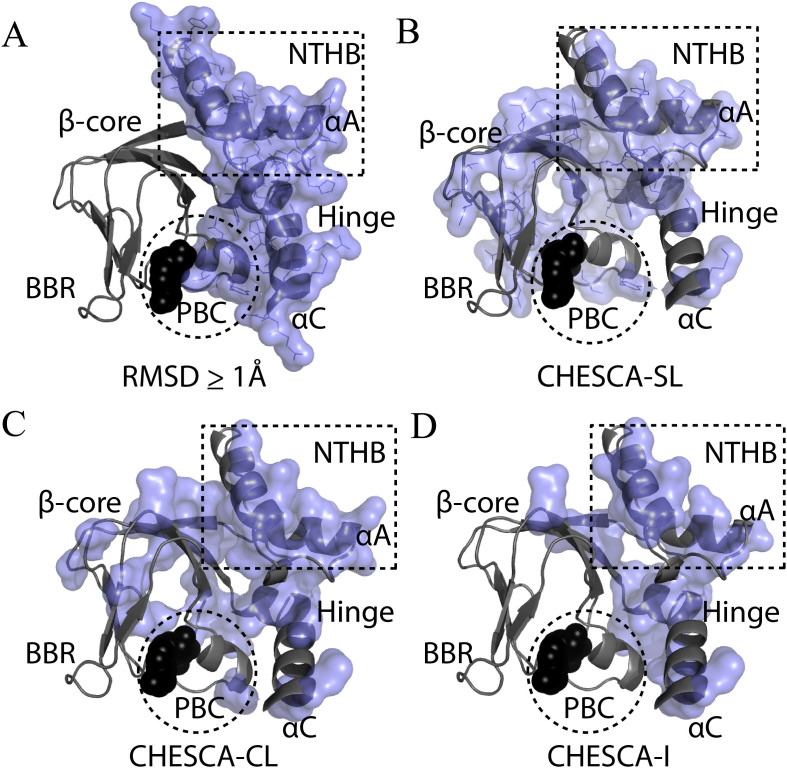
Summary maps of CHESCA-based allosteric ensembles identified for PKA RIα CBD-A. (A) Structure-based allosteric residues for the folded CBD-A. Residues with a local RMSD between the C-bound and cAMP-bound crystal structures of PKA R (PDB IDs: 3FHI and 3PNA) greater than 1 Å are shown as a blue surface. (B–D) Allosteric residues from the CHESCA-SL, -CL and -I analyses of PKA CBD-A, respectively, mapped onto its crystal structure. cAMP is shown as black spheres.

In order to further gauge the reliability of the different CHESCA options outlined here, we sought to compare the allosteric networks derived through CHESCA with those established through traditional structural comparisons. Although CHESCA offers the additional advantage of accurately mapping allosteric networks for partially unstructured regions, the comparative structural analyses of the inactive *vs.* active states for well-structured globular regions, such as the CBDs of PKA, provide a reliable CHESCA-independent map of allosteric networks, which is useful to cross-validate the CHESCA results^13^,^48^,^49^. For this purpose, residue-specific RMSD values were measured between the active (*i.e.* cAMP-bound) and inactive (*i.e.* C-subunit bound) structures of PKA RIα CBD-A^17^,^18^ and residues with RMSDs greater than or equal to 1 Å (*i.e.* 10% of the maximum RMSD) were mapped onto the cAMP-bound crystal structure^8^,^18^ to highlight the regions that undergo major structural changes during activation (Figure 6A). The majority of these residues occur in the α-subdomain, spanning the NTHB and hinge helices as well as the PBC region (Figure 6A).

The RMSD-based allosteric cluster of Figure 6A was compared to that obtained through the traditional CHESCA-SL analysis (Figure 6B), which relies on single linkage clustering. The two allosteric clusters are comparable at the level of the α-subdomain (Figure 6A,B), but in the case of the single-linkage CHESCA-SL cluster (Figure 6B) there are also several residues identified in the β-subdomain. Although the β-subdomain structure is to a large extent invariant upon cAMP-binding, selected residues from the β-core are indeed expected if they are in the vicinity of the α-subdomain and experience local changes in their spatial environment upon transition from the inactive to the active state. Additional correlations involving the β-core may also be anticipated if they play a significant role in the activation of PKA, but arise from structural and/or dynamical perturbations that fall below the resolution of changes detectable by crystallography. For example, several loops in the β-core play important roles in ligand binding and activation^5^,^50^, but due to their dynamic nature they are poorly structurally defined. Nonetheless, these explanations cannot account for all β-core residues observed in Figure 6B. For instance, single-linkage clustering detects several residues far from the α-subdomain and in rigid β-strand regions (Figure 6B). Such residues are clearly false positives caused by the chaining effects intrinsic to the single linkage clustering of CHESCA-SL.

The β-core residues identified as false positives in the single linkage CHESCA-SL are partially removed by the complete-linkage CHESCA-CL (Figure 6C). As shown in Figure 6C, CHESCA-CL still captures the α-subdomain and the β-core residues that are either in loops or adjacent to the α-subdomain, as anticipated. Very few residues from the PBC were captured, but this is expected since unique PBC perturbations from each of the different cAMP analogs induce ligand specific nearest-neighbour chemical shift changes, which compromise the linearity in at least some of the chemical shift correlations. Finally, the β-core residues identified as false positives in the CHESCA-SL are almost completely removed by the CHESCA-I (Figure 6D). Most of the α-subdomain is still captured as well as adjacent β-core residues (Figure 6D), but the allosteric cluster is now significantly sparser and some regions that were expected to generate CHESCA correlations were absent altogether (*i.e*. residues 148–151 in the NTHB and residues 168,169 and 171–174 in the β2-β3 loop). As a further validation, the proposed CHESCA methods were also applied to EPAC (Figure S6 and supplementary text). Overall, in both PKA R and EPAC the allosteric networks mapped by CHESCA-CL are consistent with those independently defined through comparative structural and/or mutational analyses of the well folded globular domains, while at the same time also capturing dynamic inhibitory sites that would have otherwise remained elusive.

### Concluding Remarks

The CHEmical Shift Covariance Analysis (CHESCA) relies on a library of perturbations with known functional profiles to comprehensively map at residue resolution allosteric networks. The CHESCA method is especially useful for identifying allosteric residues belonging to dynamic regions, such as linkers and loops, which may remain elusive to traditional structure determination methods. Here, we have identified two sources of false positives in the detection of allosteric residues through the original CHESCA algorithm (*i.e.* CHESCA-SL). One source of false positive is single linkage clustering, which tends to cluster together poorly correlated residues exhibiting different responses to the CHESCA library (‘chaining effects'). Another cause of false positives is the use of combined ^1^H and ^15^N chemical shifts, as residues with different ^1^H and ^15^N chemical shifts may result in similar combined ppm values (‘projection compression'). We have shown that both types of false positives are effectively identified using the CHEmical Shift Projection Analysis (CHESPA). The CHESPA signature that reveals false positives from the CHESCA analysis, is the presence of outlier residues with fractional activations that differ markedly from the majority of other residues in the allosteric cluster.

We have proposed two new CHESCA methods to minimize the presence of false positives. One method (*i.e.* CHESCA-CL) is based on complete rather than single linkage clustering, while the other (*i.e.* CHESCA-I) relies also on the use of separate rather than combined ^1^H and ^15^N chemical shifts. Both CHESCA-CL and -I complement the original CHESCA-SL scheme. CHESCA-SL provides an effective approach for the comprehensive detection of networks of residues involved in conformational equilibria underlying allostery. Although CHESCA-SL may lead to false positives due to chaining effects, it reduces the chances of false negatives especially for systems in which the chemical shift correlation matrix **R** is sparse. As the stringency of the analysis is increased by imposing complete linkage clustering (CHESCA-CL), the number of false positives arising from chaining effects is drastically reduced and the correlations appear to be more directed towards the core groups of residues involved in allostery. If the allosteric residues identified by CHESCA-CL still include residual false positives, as revealed by markedly different residue-specific fractional activations measured through CHESPA, the CHESCA-I is available to minimize the risk of projection compression.

**Figure 7:**
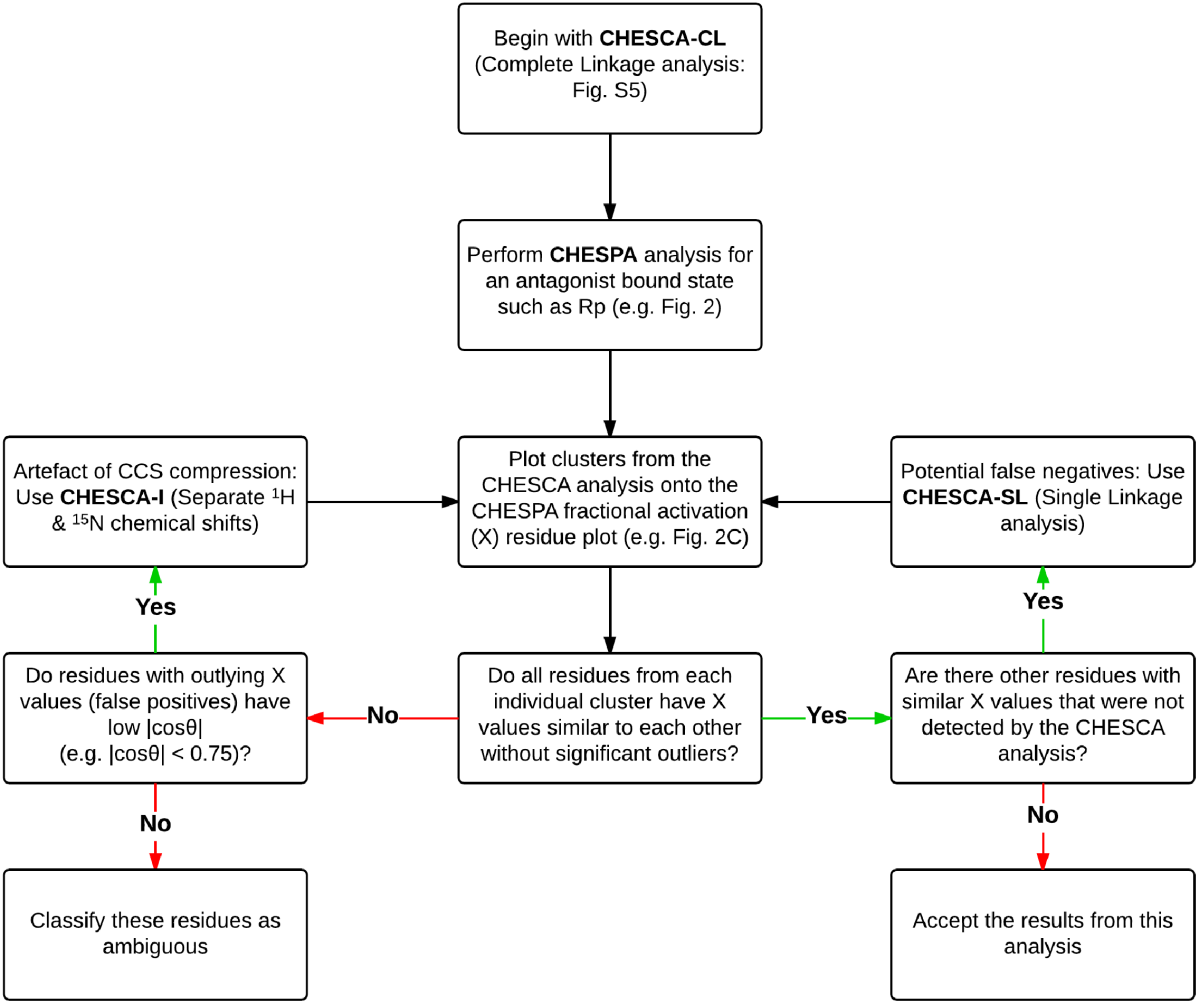
A flow chart ‘user guide' for the implementation of the proposed CHESCA tool set. Initially, the chart starts with CHESCA-CL, since it provides an optimal balance between the minimization of false positives and false negatives. If residual false positives are detected through CHESPA, the application of the CHESCA-I approach is recommended to minimize those arising from the CCS compression. If false negatives are revealed through CHESPA, the implementation of the CHESCA-SL algorithm is also advised. It is sufficient to implement each type of CHESCA analysis only once. However, multiple types of CHESCA analyses (*i.e.* SL, CL, I) are often needed for a single chemical shift data set.

CHESCA-I offers the highest level of stringency, which is advantageous as it not only reduces false positive artefacts, but it also ‘zooms in' on core allosteric residues in the protein assisting the prioritization of sites to be tested by mutagenesis. However, the added stringency of CHESCA-I also results in increased false negatives. In this respect, a balanced application of the CHESCA method is likely to be the CHESCA-CL. However, it is important to consider that CHESCA-CL is part of a larger tool set of complementary chemical shift analyses (*i.e*. CHESCA–SL, -CL and -I as well as CHESPA) and it is advised that multiple analyses from this tool set be implemented for a single chemical shift data set in order to obtain a reliable reconstruction of the underlying allosteric networks. A ‘user guide' for the proposed CHESCA tool set is summarized by the flow chart shown in Figure 7.

## Methods

The { ^15^N, ^1^H}-HSQC datasets and chemical shifts used for PKA RIα CBD-A (91-244) were acquired at a protein concentration of 10 μM and a ligand concentration of 3 mM^12^. The error bars were derived from the standard deviation of chemical shifts at saturation, *i.e.* ligand concentrations of 1-3 mM. The EPAC1_h_ (149-318) chemical shifts were as in Selvaratnam *et. al.*^25^*.* The protocol for CHESCA-SL and CHESPA have been described elsewhere^40^. Further details on the methods are available as Supplementary Material.

## Author Contributions

S.B., M.A., R.S., A.B., and G.M. designed research; S.B., M.A., R.S., A.B. performed research; S.B. and G.M. analyzed data; and S.B. and G.M. wrote the paper. All authors reviewed the manuscript.

## Supplementary Material

Supplementary Information Supplementary Information

## Figures and Tables

**Figure 1:**
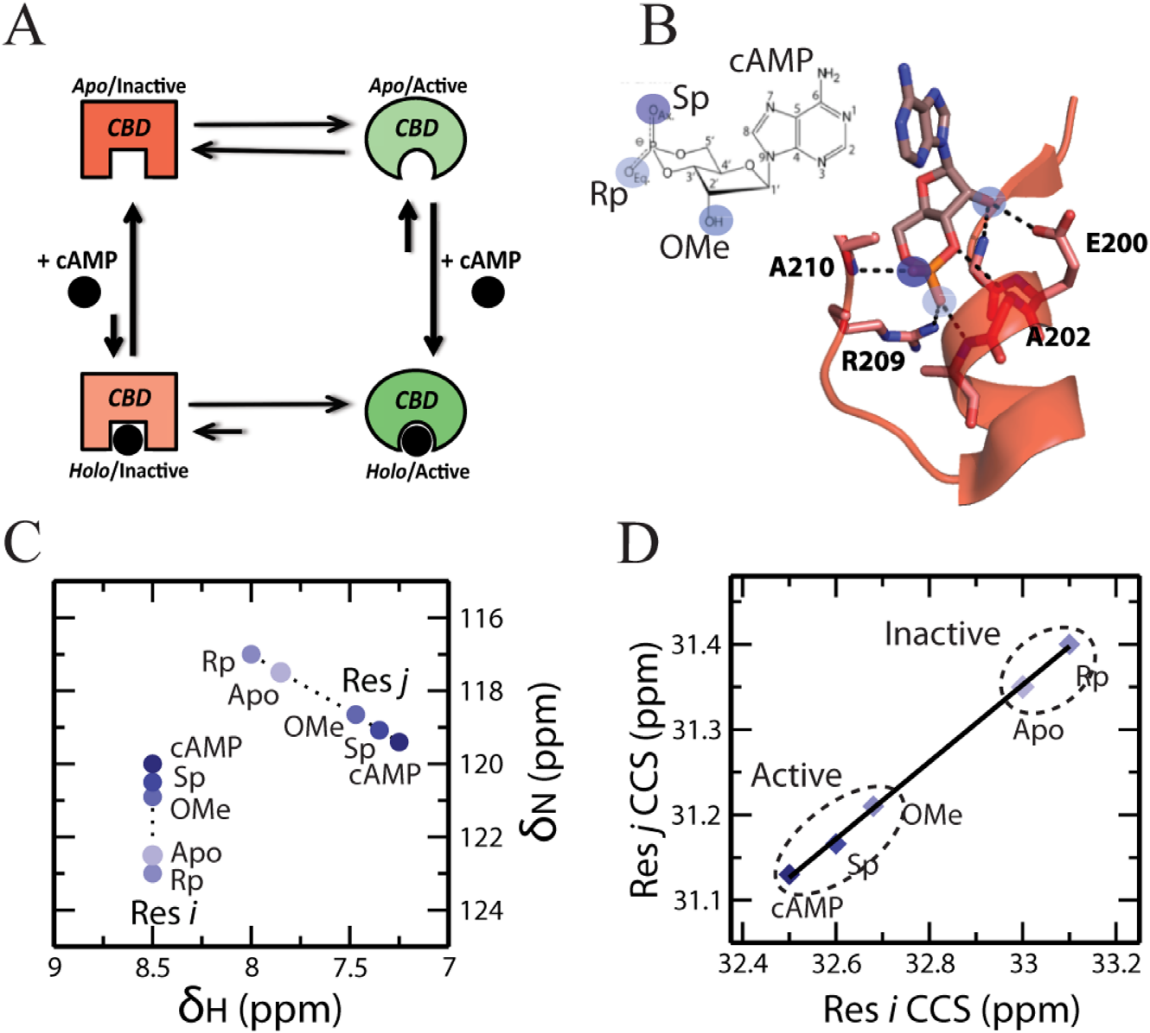
Allosteric thermodynamic cycle and the CHESCA experimental design. (A) Allosteric thermodynamic cycle of PKA RIα based on the coupling of auto-inhibitory and cAMP-binding equilibria. (B) Chemical library selection for the CHEmical Shift Covariance Analysis (CHESCA). The blue circles mark cAMP sites that interact directly with the CBD and are modified in the CHESCA library (*e.g.* S_p_-cAMPS, R_p_-cAMPS and 2′-OMe-cAMP). (C) Representative HSQC peaks of two residues, *i* and *j*, subject to the CHESCA perturbations outlined in (B). (D) The corresponding pairwise inter-residue correlation plot for the compounded chemical shifts (CCS) of residues *i* and *j*.
