# Supplementary material for: A Tool Set to Map Allosteric Networks through the NMR Chemical Shift Covariance Analysis
Source: Sci Rep. 2014 Dec 8;4:7306. doi: 10.1038/srep07306 (PMC4258684; doi:10.1038/srep07306)
Supplement: Supplementary Information [file srep07306-s1.pdf]

Supplementary Material

for

A Tool Set to Map Allosteric Networks through the NMR Chemical Shift Covariance Analysis

Stephen Boulton[1], Madoka Akimoto[2], Rajeevan Selvaratnam[2], Amir Bashiri [2]
and Giuseppe Melacini[*1,2]

[1]Department of Biochemistry and Biomedical Sciences
[2]Department of Chemistry and Chemical Biology
McMaster University, 1280 Main Street West, Hamilton, Ontario, L8S 4M1, Canada

*To whom correspondence should be addressed: melacin@mcmaster.ca

Supplemental Methods

CHESPA Analysis and Fractional Activations of PKA RIα (91-244). Briefly, the CHESPA analysis relies on {^{15}N,^{1}H}-HSQC spectra acquired for three samples of the protein of interest. In the case of PKA RIα (91-244), CHESPA focused on the *apo*, cAMP-bound and R_p-cAMPS-bound samples under fully saturating conditions. Two CHESPA vectors are then defined: a reference vector for the endogenous allosteric effector (*i.e.* vector **B** in Figure S2) and a vector for the perturbed state (*i.e.* vector **A** in Figure S2) to be compared to the reference state. The magnitude of vector **B** measures the 'distance' between the *apo* and cAMP-bound peaks calculated for each residue using the equation:

$$\Delta\delta_{NHcomb} = \sqrt{[(\delta_{H,apo} - \delta_{H,cAMP})^2 + (0.2*(\delta_{N,apo} - \delta_{N,cAMP}))^2]} \qquad (5)$$

The distance between the *apo* and R_p-cAMPS bound states was also calculated and defined as the magnitude of vector **A** in the CHESPA analysis. The angle between the **A** and **B** vectors is denoted as θ so that:

$$\cos\theta = \frac{\vec{A}\bullet\vec{B}}{|\vec{A}||\vec{B}|} \qquad (6)$$

and the normalized projection of **A** onto **B** is defined as the 'fractional activation' (X), which is calculated as:

$$X = \frac{\vec{A}\bullet\vec{B}}{|B|^2} \qquad (7)$$

The fractional activation reveals whether a given perturbation (*e.g.* Rp-cAMPS) shifts the protein towards activation ($X > 0$) or inactivation ($X < 0$).

CHESCA Analyses of PKA RIa CBD-A (91-244) and EPAC1$_h$ (149-318). The CHESCA protocol has been described elsewhere [3,5,6]. Briefly, CHESCA relied on {^{15}N,^{1}H}-HSQC spectra acquired for the *apo* and four *holo* states (cAMP, R_p-cAMPS, S_p-cAMPS and 2'-OMe-cAMP) under fully saturating conditions. Spectra were assigned and the combined chemical shifts (CCS) for each residue were calculated using equation (1). The inter-residue CCS pairwise correlations in the correlation matrix (**R** matrix) with an absolute value of the Pearson's correlation coefficients greater than a selected threshold (*e.g.* 0.95-0.98) were selected. Hierarchical agglomerative clustering (AC) of correlated residues was performed using the Cluster 3.0 software[7] and visualized as dendrograms with Java TreeView[8]. Singular value decomposition analysis of HSQC chemical shifts was performed using Octave.

Structure-Based Allostery Predictions for PKA RIα (91-244). Local RMSD calculations were performed in MolMol[9] using the PDB structures for C-bound PKA (PDB ID 3FHI)[1] and cAMP-bound PKA (PDB ID 3PNA)[2]. The structures were aligned at their β-cores (150-225) and the local RMSDs were measured for each residue in the structured region spanning residues 112-236. The surface of the residues affected by the cAMP-dependent allosteric conformational change (*i.e.* with RMSD > 1 Å, which is 10% of the maximum RMSD) was mapped onto the structures using Pymol (http://www.pymol.org).

Validation of the EPAC CHESCA Analysis

In Figure S6 the residues from the allosteric clusters in each CHESCA analysis are mapped in green in both the chemical shift plots and the cAMP-bound crystal structure of EPAC[4]. Previously, using the single linkage based CHESCA-SL (Figure S6A,B), we identified an allosteric network that stemmed from the PBC and extended to the α4 and α6 helices *via* a series of hydrophobic spines, culminating at the inhibitory ionic latch in the NTHB (Figure S6A,B). The inhibitory function of the ionic latch (IL) of EPAC was independently supported by site-directed mutagenesis[10]. However, single-linkage clustering also incorporated a number of residues in the β-subdomain region that were not expected to participate in the allosteric network based on the invariance of the β-core[3,10] (Figure S6A,B). When the complete-linkage CHESCA-CL was applied, several unexpected residues from the β-core were removed, while residues that connected the PBC with the ionic latch were still detected, as expected (Figure S6C,D). The results from the complete-linkage $^{15}N/^{1}H$ intersection CHESCA-I analysis (Figure S6E,F) were in general similar to the complete-linkage CCS clustering (Figure S6C,D), but with the PBC residues now missing.

References

1. Kim, C. Xuong, N.-H. & Taylor, S. S. Crystal structure of a complex between the catalytic and regulatory (RIalpha) subunits of PKA, *Science* **307,** 690–696 (2005).

2. Badireddy, S. *et al.* Cyclic AMP analog blocks kinase activation by stabilizing inactive conformation: conformational selection highlights a new concept in allosteric inhibitor design, *Mol. Cell Proteomics* **10,** M110.004390 (2011).

3. Selvaratnam, R. Chowdhury, S. VanSchouwen, B. & Melacini, G. Mapping allostery through the covariance analysis of NMR chemical shifts, *Proceedings of the National Academy of Sciences* **108,** 6133–6138 (2011).

4. Rehmann, H. *et al.* Structure of Epac2 in complex with a cyclic AMP analogue and RAP1B, *Nature* **455,** 124–127 (2008).
5. Selvaratnam, R. Mazhab-Jafari, M. T. Das, R. Melacini, G. & Hofmann, A. The Auto-Inhibitory Role of the EPAC Hinge Helix as Mapped by NMR, *PLoS ONE* **7,** e48707 (2012).
6. Akimoto, M. *et al.* Signaling through dynamic linkers as revealed by PKA, *Proceedings of the National Academy of Sciences* **110,** 14231–14236 (2013).
7. de Hoon, M J L, Imoto, S. Nolan, J. & Miyano, S. Open source clustering software, *Bioinformatics* **20,** 1453–1454 (2004).
8. Saldanha, A. J. Java Treeview--extensible visualization of microarray data, *Bioinformatics* **20,** 3246–3248 (2004).
9. Koradi, R. Billeter, M. & Wuthrich, K. MOLMOL: a program for display and analysis of macromolecular structures, *J Mol Graph* **14,** 51-5, 29-32 (1996).
10. Rehmann, H. Das, J. Knipscheer, P. Wittinghofer, A. & Bos, J. L. Structure of the cyclic-AMP-responsive exchange factor Epac2 in its auto-inhibited state, *Nature* **439,** 625–628 (2006).

Supplemental Figures

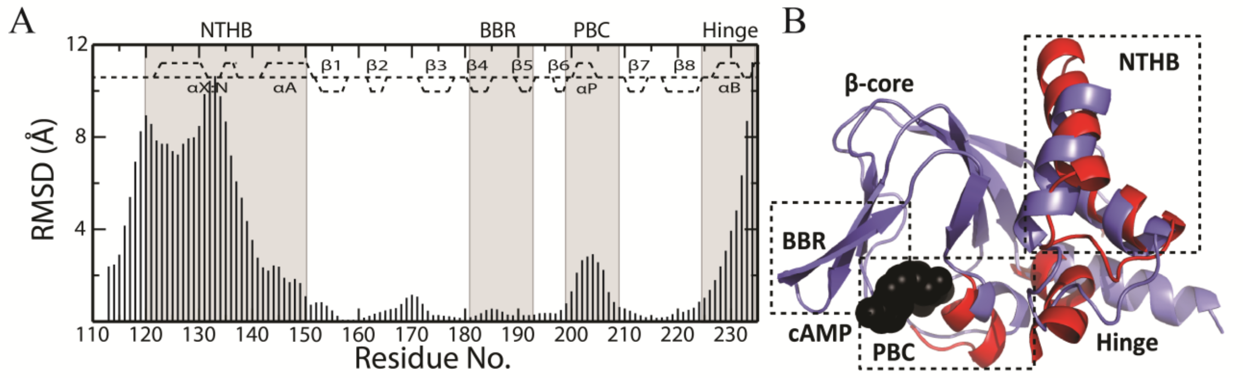

Figure S1: *Allosteric cAMP-dependent structural changes in the well folded cAMP-Domain A of PKA RIα (91-244) as revealed by RMSDs*. **(A)** RMSDs of individual residues measured between the structures of C-subunit bound PKA RIa CBD-A (PDB code 3FHI)[1] and cAMP bound PKA (PDB code 3PNA)[2] aligned at their β-cores (residues 150-225), as shown in panel (B). The dashed lines along the top represent the secondary structure of the protein. **(B)** An overlay of the crystal structures for C-bound (blue) and cAMP-bound (red) PKA aligned at their β-cores. cAMP is shown as black spheres.

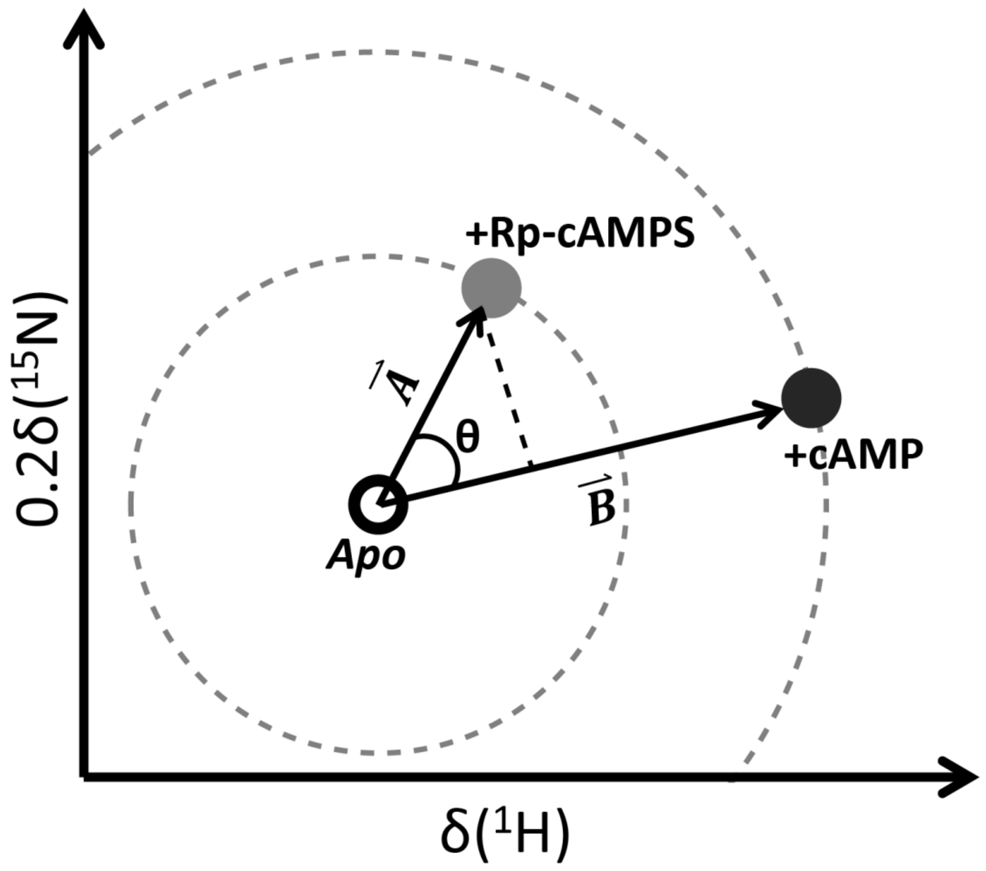

Figure S2: *Schematic representation of the vectors utilized in the CHEmical Shift Projection Analysis (CHESPA).* The small solid circles represent HSQC peaks for the *apo* (Black, Open), R_p-cAMPS (Rp) bound (Gray, Closed) and cAMP bound (Black, Closed) states. The $\Delta\delta_{NHcomb}$ between the Rp -bound and *apo* states was calculated as the magnitude of vector **A**. Similarly, the $\Delta\delta_{NHcomb}$ between the cAMP-bound and *apo* states was calculated as the magnitude of vector **B**. The angle between vectors **A** and **B** is defined as θ (Equation 6) and the fractional activation (X) is measured by projecting vector **A** onto vector **B** and normalizing the projection to the magnitude of vector **B** (Equation 7).

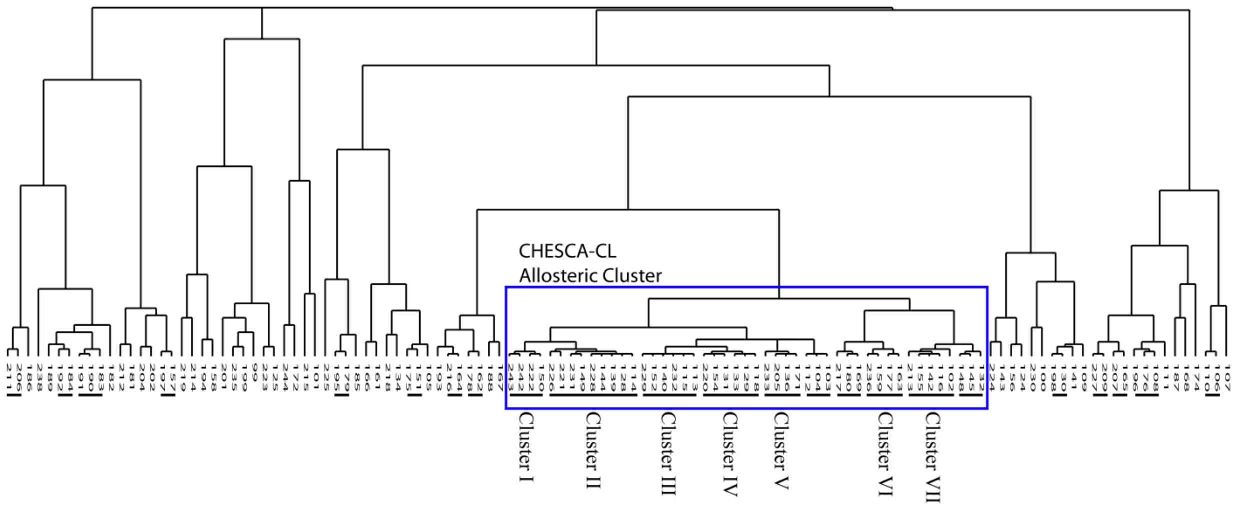

Figure S3: *Whole dendrogram representing the complete-linkage agglomerative clustering of PKA RIα (91-244).* The horizontal lines beneath the dendrogram represent individual fragmented clusters that were computed from complete-linkage clustering using a cutoff of 0.98 for the absolute value of the correlation coefficient. Out of these clusters, seven include more than three residues. These seven clusters are further analyzed in Figure S4 and the smallest branch in the complete dendrogram including them is marked by a blue rectangle. All residues within this branch fit the criteria that define them as a single network (Figure S5) and were therefore reconstituted into a single allosteric cluster.

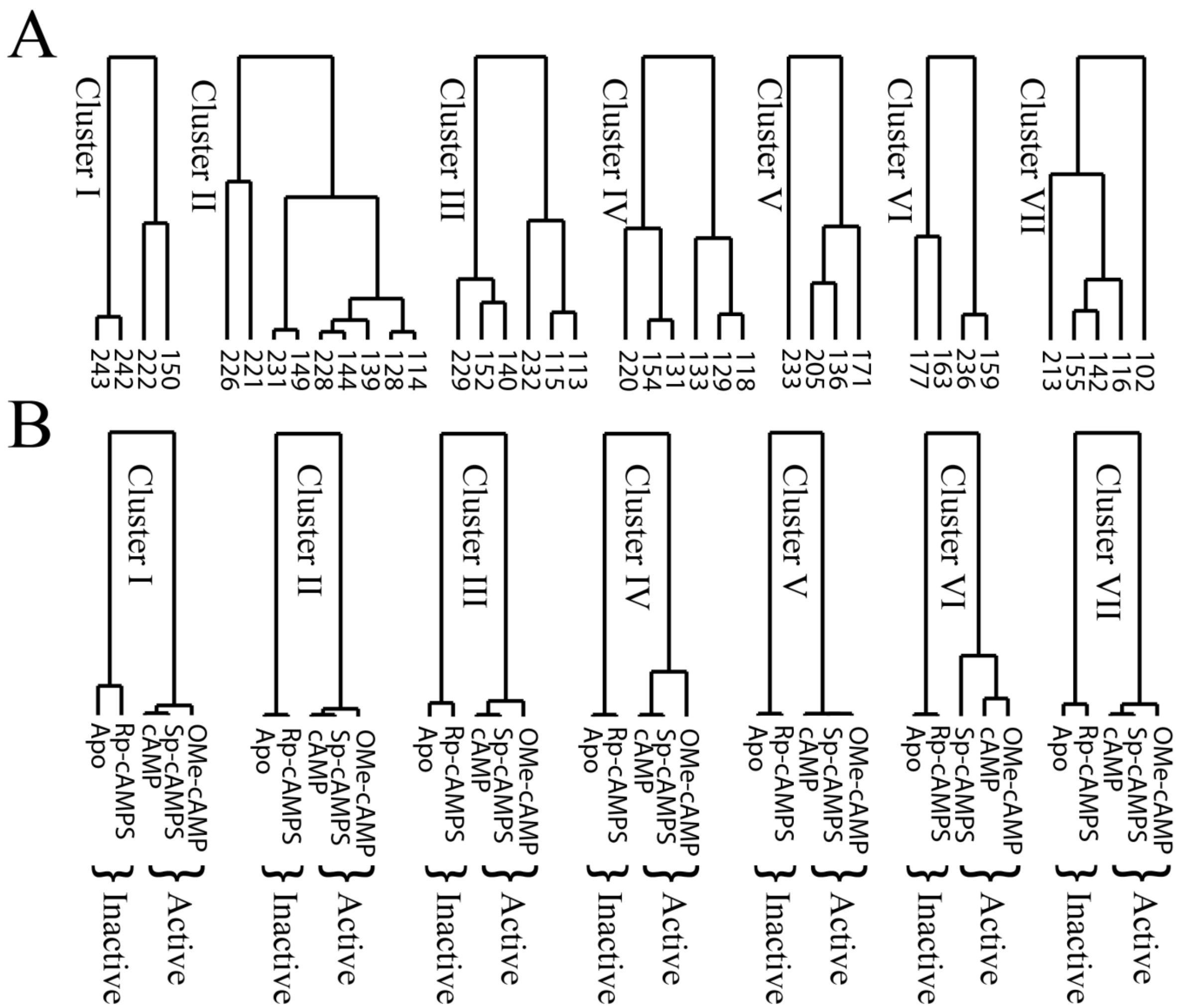

Figure S4: *Complete-linkage clustering results in fragmentation of the allosteric cluster into functionally similar sub-clusters*. **(A)** Residue-based dendrograms generated through complete linkage clusters. The branch height is proportional to the correlation coefficients between clusters of residues. **(B)** State-based dendrograms of the clusters shown in **(A)**. Even though complete-linkage clustering results in the formation of several new sub-clusters, they all share similar functional state distributions (*i.e.* inactive states: *apo* and Rp-cAMPS, separated from the active states: cAMP, Sp-cAMPS and 2’-OMe-cAMP).

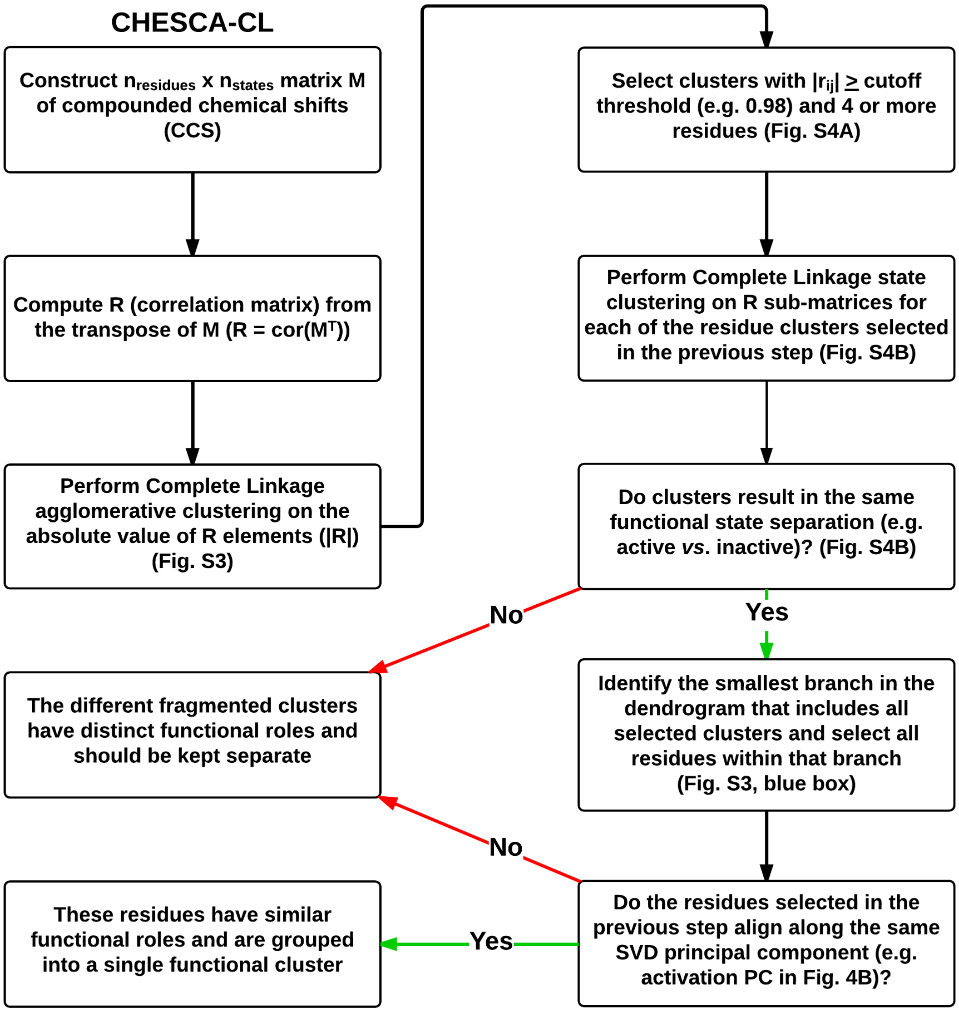

Figure S5: *A protocol for the implementation of the CHESCA-CL analysis including algorithms for the reconstruction of allosteric clusters starting from fragmented sub-clusters generated by complete-linkage*. Selection of allosteric clusters from complete-linkage clustering is achieved by first selecting groups with more than three residues and a Pearson's correlation coefficient ($|r_{ij}|$) ≥ 0.98 (Figure S4A). If the groups from the previous step have the same functional separation (*i.e.* active *vs.* inactive) in the respective state dendrograms (Figure S4B), the smallest branch of the complete-linkage dendrogram that spans them is identified (blue box in Figure S3) and all residues in this branch are grouped together. If all residues within this group align along the same functional PC (*i.e.* activation in Figure 4B) from the SVD analysis, they are defined as a single allosteric cluster. Correlation and singular value decomposition (SVD) computations are performed similarly to Selvaratnam *et al.* [3].

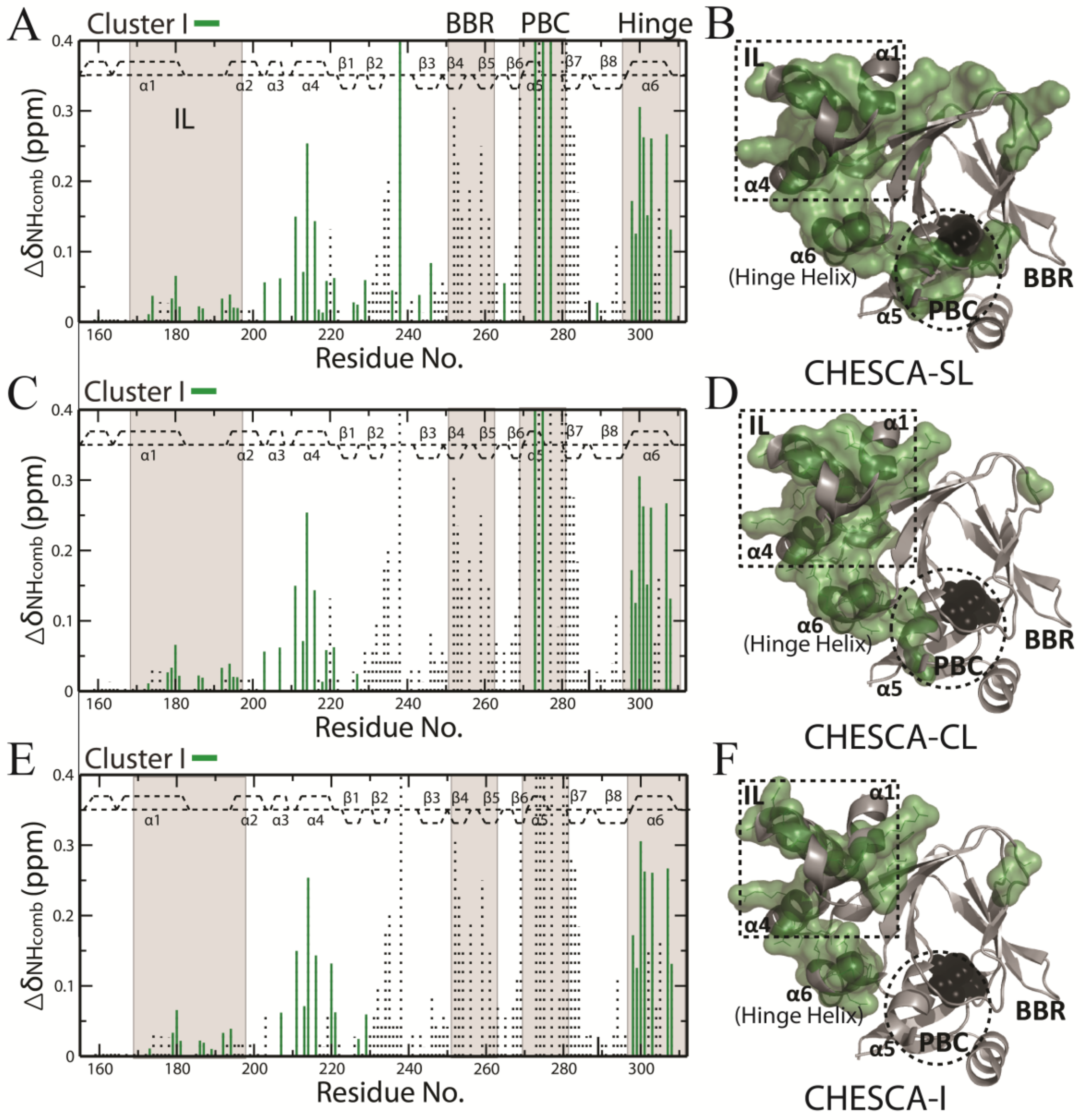

Figure S6: *CHESCA toolset applied to EPAC1$_h$.* **(A)** CCS plot for cAMP binding to EPAC1 (149-318) with residues from the CHESCA-SL allosteric cluster displayed as solid green lines. **(B)** Green residues from panel (A) mapped onto the crystal structure of the homologous EPAC2 (PDB ID: 3CF6)[4]. Dashed lines mark the ionic latch (IL), critical for EPAC auto-inhibition, and the Phosphate Binding Cassette (PBC), where cAMP binds. **(C)** CCS plot for cAMP binding to EPAC with residues from the CHESCA-CL allosteric cluster displayed as solid green lines. **(D)** Green residues from panel (C) mapped onto the crystal structure of EPAC2. **(E)** CCS plot for cAMP binding to EPAC with residues from the CHESCA-I allosteric cluster displayed as solid green lines. **(F)** Green residues from panel (E) mapped onto the crystal structure of EPAC2.
